# Early Improper Motion Detection in Golf Swings Using Wearable Motion Sensors: The First Approach

**DOI:** 10.3390/s130607505

**Published:** 2013-06-10

**Authors:** Sara Stančin, Sašo Tomažič

**Affiliations:** Faculty of Electrical Engineering, University of Ljubljana, Tržaška cesta 25, Ljubljana 1000, Slovenia; E-Mail: saso.tomazic@fe.uni-lj.si

**Keywords:** golf swing, wearable motion sensors, motion deviations, improper motion, early phase motion analysis, principal components analysis

## Abstract

This paper presents an analysis of a golf swing to detect improper motion in the early phase of the swing. Led by the desire to achieve a consistent shot outcome, a particular golfer would (in multiple trials) prefer to perform completely identical golf swings. In reality, some deviations from the desired motion are always present due to the comprehensive nature of the swing motion. Swing motion deviations that are not detrimental to performance are acceptable. This analysis is conducted using a golfer's leading arm kinematic data, which are obtained from a golfer wearing a motion sensor that is comprised of gyroscopes and accelerometers. Applying the principal component analysis (PCA) to the reference observations of properly performed swings, the PCA components of acceptable swing motion deviations are established. Using these components, the motion deviations in the observations of other swings are examined. Any unacceptable deviations that are detected indicate an improper swing motion. Arbitrarily long observations of an individual player's swing sequences can be included in the analysis. The results obtained for the considered example show an improper swing motion in early phase of the swing, *i.e.*, the first part of the backswing. An early detection method for improper swing motions that is conducted on an individual basis provides assistance for performance improvement.

## 1. Introduction

Performing the golf swing is a demanding process that involves the entire psychophysical system [1–6]. It is one of the most difficult biomechanical motions to execute in sports, given the challenging performance requirement to swing a relatively long club at a relatively small ball with maximal velocity [1]. The fundamental purpose of the swing is to develop sufficient force and align the clubface to be square to the target at the moment of striking the ball with the club so the ball can be sent directly towards the target [2]. To achieve this goal, the golfer must perform a number of mechanical feats resulting in the coordinated movement of all involved body segments. The golfer's motion determines the motion of the golf club, the clubhead velocity, position, direction, and orientation at the point of striking the ball, and consequently the motion of the ball, including its flight direction and carry distance. Expressed in succinct words by Hank Hayne, one of golf's well-known instructors, “making a great swing, one will hit a great shot” [3].

Shot consistency and accuracy in terms of direction and distance are fundamental elements of performance in golf. According to another well-known golf coach, John Jacobs, the method employed to swing the club and hit the ball is actually of no importance: “The only purpose of the golf swing is to move the club through the ball square to the target at maximum speed. How this is done is of no importance at all, so long as the method employed enables it to be done repetitively” [5]. Because of the demanding high level of skill and attention, even after obtaining a certain degree of proficiency, most amateur golfers still lack consistent performance. During practice sessions at the driving range, the golfer observes the flying characteristics of the ball and tries to relate the shot outcome results to his or her swing movements. However, after performing a series of successful shots, the golfer is often puzzled at not being able to identify the improper motion resulting in a non-intended ball flight.

To analyse the swing and provide feedback information necessary for golf performance improvement, it is common to refer to low-handicap golfers as a template for appropriate swing motion [6–9]. However, despite the fact that the general geometry and physics of the golf swing are known and have been widely elaborated [2,4], individual performance varies due to the complexity of the golf swing, depending on a number of decisive parameters, including the player's physical ability, natural predisposition, and physiognomy, as well as their personal approach [10–13]. Swing performance differences of equally skilled professional players have also been reported [14]. All of the aforementioned factors suggest that a universal perfect swing, common for all players, does not exist. Providing a golf swing motion analysis that would consider the player's individual characteristics would contribute to a better and more comprehensive swing motion evaluation.

In the area of modern methods of motion tracking, relevant data are the starting point for comprehensive motion analysis. Wearable motion sensors provide data that directly reflect the motion of individual body parts and can enable the development of advanced tracking and analysis procedures [15–18]. Microelectromechanical system (MEMS) motion sensors are small, light, widely affordable, and come with their own battery supply; they are extremely suitable for tracking the motion of a golf swing. These sensors cause minimal physical obstacles for swing performance and can provide simple, repeatable, and collectible golf swing data at any outdoor driving range; furthermore, they are well suited for usage on the game course itself. In this manner, the collected motion data can be supplemented with other authentic descriptive shot information, such as ball displacement. This contrasts most biomechanical research involving the golf swing, which has been conducted in laboratory-type situations and has used proxy measurements [19].

MEMS motion sensors offer the possibility of collecting data on golf swing dynamics. A number of recent golf swing analyses offer insight into how specific parameters, such as amplitude and timing data of the peak segment speed, affect the golf shot flight direction, carry distance, and consistency [20,21]. However, exploring golf swing dynamics enables a more comprehensive analysis and can offer a greater understanding of the effects of proper or improper motions on golf swing performance.

In aiming for an identical shot outcome in multiple trials, a particular golfer would attempt to perform the swing in an identical manner, resulting in absolutely consistent shots. In reality, some deviations from the desired swing motion are always present due to the high level of technical difficulty. It is highly unlikely that a number of coordinated rotational and translational motions of all body segments utilised in the swing are repeatedly performed identically. To achieve a good shot, the golf clubhead must arrive at the ball with the correct velocity, position, direction, angle of attack, and orientation. However, while the absolute invariance of the aforementioned parameters at the point of striking the ball is highly desirable, the influence of motion data variability to this point of the swing has not yet been thoroughly investigated. When considering motor equivalence, a number of different motion patterns exist that would still allow for the same outcome [22], and thus, not all golf swing performance deviations are detrimental to performance.

The aim of the research presented in this paper is to explore the individual swing motion to differentiate acceptable deviations, (*i.e.*, those not having an effect on swing accuracy and consistency) from those leading to unsuccessful shots, thus enabling the detection of an improper swing motion. The intention is to provide for simple and practical detection of improper motion. To accomplish this task, multiple swing motion data are captured using a wearable motion sensor that is comprised of MEMS gyroscopes and accelerometers. Each swing observation is labelled according to its performance. Along with objective outcome evaluations, subjective marks provided by the golfer are also considered for the overall performance evaluation. Reflecting the overall feeling and easiness of swing motion, the subjective marks are very valuable when considering the player's individual swing characteristics. A reference set of properly performed individual swings is formed based on the collected data and the considered swing observation labels. The successful repetition of these swings is the golfer's essential practice aim. Due to the importance of the consistency of the golf swing tempo, all reference swings should have the same timing.

Among different approaches tried, principal component analysis (PCA) [23,24] gave the best results. By exploring similarities and dissimilarities in discrete data, PCA permits for a separation of proper golf swing components from improper motion.

Using PCA, the reference observations of the properly performed swings are used to obtain the desired swing motion and are further analysed. The resulting set of PCA components forms the basis of the acceptable motion deviations from the desired motion. This basis is used to analyse other swing observations and improper motion detection. Such an analysis is made under the assumption that the set of properly performed swings encompass all acceptable deviations of a proper golf swing motion such that any deviation of the analysed swings not covered with the reference PCA components likely belongs to an improper golf swing motion.

The proposed method refers to a particular swing with a particular club and in a particular environment. Hence, for different clubs, shots and environments, a different reference set of properly performed swing should be constructed. In this way, the appropriate PCA components for a concrete swing can be considered.

Any portion of the golf swing (e.g., only the backswing) can be analysed, which enables the detection of an improper motion in the early phases of the swing. As John Jacobs asserts, golf is a reaction game: “Do one thing right in the golf swing and it will lead to another right. Do one thing wrong and it will produce another wrong” [5]. In the best-case scenario, when a wrong motion is present, a number of compensatory movements will have to be included to correct the position of the golf club at the point of its impact with the ball. With early improper motion detection, focus is given to the problem itself and not to the subsequent reactions. Detecting an improper swing motion in the early phases of the swing is essential for the offline performance improvement process and for enabling feedback applications that can help improve shot accuracy and consistency in real time.

If upgraded with sufficient processing power, wearable motion sensors can be used to perform well-designed real-time analysis of the collected data. If further equipped with adequate small and light hardware (for example, audio speakers), useful feedback applications could be enabled. It has been reported that the usage of an auditory feedback channel permits one to improve swing performance in a continuous learning process [25]. Instantaneously providing feedback information and bringing it to consciousness allows “one to directly *sense* and *feel* actions that lead to successful and unsuccessful swing sequences” [25].

This paper is organised as follows: in Section 2, we introduce the analysis of the golf swing deviations from the desired motion based on PCA. The measurements are described in Section 3, and the results of the proposed analysis are presented in Section 4. In Section 5, we draw conclusions and provide directions for further work.

## 2. Detection and Analysis of Improper Motion in a Golf Swing

The analysis proposed in this paper can be conducted based on the time sequences of different kinematic data, such as the angular velocity or acceleration of a specific body segment. Analysed observations can include entire swing motion sequences or swing segments (*i.e.*, backswings). All observations are labelled according to their performance as proper or improper.

In the following analysis, all matrices are denoted in upper bold case. Vectors are denoted in lower bold case. Scalars are denoted in upper and lower italic case.

As a thorough insight into PCA is outside the scope of this paper, a formally succinct description is presented in the following text, while the primary focus is given to its application for improper golf swing motion detection.

### 2.1. Principal Components of Reference Observation Deviations

From a set of *N* properly performed swings, we obtain *N* reference observations. All observations are represented with column vectors of equal length *L*. Ideally, all of the considered swings that are performed properly would be performed entirely identically, and the analysed data would be equal among all observations. Realistically, the observations deviate from their mean. These deviations are clearly non-detrimental to performance and are thus treated as acceptable. The mean value of all reference observations is treated as the desired swing motion represented with a column vector **d**. Deviations of the reference observations from the desired swing motion are represented with an *L* × *N* matrix **R**. We later refer to **R** as the set of acceptable deviations. The *n*-th column of **R**, denoted **r***_n_*, thus represents the deviation of the *n*-th observation from the desired motion **d**. It is to be noted that, due to possibly present artefacts in the reference observations, the mean value **d** can represent unphysical motion. As will be seen in further text, there is no requirement for a swing to be equal to **d** to be evaluated as properly performed. Hence, in the context of the proposed method, the possibly unphysical motion in **d** is not a problem.

Following the PCA [23,24], we obtain the principal components **p***_m_* of the acceptable deviations set **R**. These principal components are vectors of length *L*. The number of principal components is equal to the rank of matrix **R**. The *m*-th principal component has an associated non-negative eigenvalue *λ_m_*. Eigenvalue *λ_m_* is equal to the variance of the acceptable deviations set **R** covered with the *m*-th principal component. These variances are direct measurements of **R** deviation from the desired motion **d**. Ideally, if all reference observations were identical, the number of principal components would be zero.

The principal components are sorted according to the corresponding *λ_m_* values in descending order. The first principal component covers for the largest amount of **R** variance. In further analysis, we tease the first *M* principal components from the remaining principal components that cover for less than *α* of **R** variance each, which can be attributed to noise and/or different artefacts. The concrete value of *α* depends on noise and artefacts and should be established empirically.

We denote the *L* × *M* matrix of the acceptable deviation set *M* principal components with **P**. Columns of **P** are the principal components **p***_m_*.

Now, we can obtain an *M* × *N* matrix of PCA coefficients **C** according to:

(1) $$\text{C}={\text{P}}^{\text{T}}\cdot \text{R}$$

Coefficient *c_m,n_* in the *m*-th row and *n*-th column of matrix **C** is equal to the cross-correlation or dot product of the *m*-th principal component **p***_m_* and *n*-th acceptable deviation **r***_n_*. In this manner, the coefficient *c_m,n_* is a measure of the principal component **p***_m_* contribution to the deviation **r***_n_*.

From the principal components **P** and coefficients **C**, we can reconstruct the approximation of the acceptable deviations set:

(2) $${\text{R}}^{\prime }=\text{P}\cdot \text{C}$$

If all of the principal components were included in **P**, then **R'** would be equal to **R**. The difference between **R** and **R'**, resulting from teasing the first *M* principal components from the components attributed to noise and/or artefacts, is the acceptable deviations set residual:

(3) $$\text{E}=\text{R}-{\text{R}}^{\prime }$$

The *n*-th column of matrix **E** is the residual deviation **e***_n_* of the *n*-th acceptable deviation **r***_n_*.

### 2.2. Improper Motion Detection

Let us now denote the deviation of the swing of interest from the desired swing **d** with a column vector **x**. The detection of an improper motion is achieved in one to three steps.

#### Step One

For a proper swing motion, we expect that the mean square value (msv) of **x** is lower or equal to the maximum acceptable msv of the reference deviations **r***_n_*:

(4) $$\text{msv}(\text{x})\leq k\max(\text{msv}({\text{r}}_{n}))$$

where *k* represents the tolerance factor. The concrete value of *k* is problem dependant and should be empirically determined. If the analysis yields too many false positives, *i.e.*, properly performed swings recognised as improper, then *k* should be increased. If the analysis yields too many false negatives, *i.e.*, improperly performed swings recognised as proper, then *k* should be decreased.

The msv(**x**) that does not fulfil Equation (4) indicates that the deviation of the swing of interest exceeds the maximum acceptable deviation, and thus, an improper motion is detected. However, a fulfilled Equation (4) does not yet indicate that the swing of interest is entirely properly performed. Improper motion can also be attributed to an excessive content covered by a particular principal component **p***_m_* or to the remaining content not covered by any of the principal components **p***_m_*.

#### Step Two

For each reference principal component **p***_m_*, we obtain a coefficient *a_m_*, *i.e.*, a cross-correlation of **x** with **p***_m_*. Denoting the column vector of components *a_m_* with **a**, we can write:

(5) $$\text{a}={\text{P}}^{\text{T}}\cdot \text{x}$$

Coefficient *a_m_* is a measure of reference principal component **p***_m_* contribution to deviation **x**. As principal components cover for acceptable motion deviations, for a properly performed swing motion, all coefficients *a_m_* should conform to:

(6) $$k\;\underset{n}{\min}(c_{m,n})\leq a_{m}\leq k\;\underset{n}{\max}(c_{m,n})$$

where *k* is the empirically determined tolerance factor. In each row of matrix **C**, the acceptable cross-correlation range is defined with maximal and minimal values of coefficients *c_m,n_*. The content of **x** covered with principal component **p***_m_* that does not exceed the maximal acceptable content covered with this particular principal component represents an acceptable motion deviation. If one or more coefficients *a_m_* are not in this acceptable range, an improper motion is detected in the swing of interest.

#### Step Three

If all coefficients *a_m_* fulfil Equation (6), then all vector **x** content that is covered with principal components **p***_m_* is within the permissible limits. The remaining content of **x**:

(7) $${\text{x}}_{\text{r}}=\text{x}-\text{P}\cdot \text{a}$$

can be partially attributed to an improper motion and partially to noise and/or different artefacts. For a properly performed swing, the msv of **x_r_** should not exceed the maximal acceptable msv of deviation residual **e***_n_*:

(8) $$\text{msv}({\text{x}}_{\text{r}})\leq k\;\text{max}(\text{msv}({\text{e}}_{n}\text{))}$$

If Equation (8) is not fulfilled, then an improper motion is detected in the swing of interest.

From the analysis presented in steps two and three, we can conclude that the concrete value of *α*, which determines the portion of **R** variance that is attributed to noise and/or different artefacts, is not crucial for the improper motion detection process. This parameter represents a compromise between precision and computational complexity. With a lower value of *α*, more principal components are included in **P** and the improper motion detection is more precise. At the same time, increasing the principal components considered in step two of the analysis creates a greater computational burden.

### 2.3. Improper Motion Analysis

Detection of an improper motion only provides information about the presence of unacceptable motion deviations in the swing of interest. If improper motion is detected, the swing of interest is evaluated as improperly performed. To obtain additional information about the detected improper motion, we must observe the residual deviations in the time domain. These residual deviations comprise unacceptable deviations, as well as noise and/or different artefacts.

To analyse the improper motion in the time domain, all three steps of the analysis must be performed, even when the improper motion is detected in the first or second steps. We obtain the residual deviations **ε** according to

(9) $$\varepsilon =\sum_{l}{\text{p}}_{l}\;(a_{l}-\text{sign}(a_{l})\underset{n}{\max}(c_{l,n}\;\text{sign}(a_{l})))+{\text{x}}_{\text{r}}$$

where *l* takes all values of indices *m* for which coefficients *a_m_* do not satisfy Equation (6). The first part of Equation (9) represents the excessive content of **x** covered with principal components **p***_l_* and **x_r_** represent the content of **x** not covered with principal components **p***_m_* according to Equation (8). If all coefficients *a_m_* fulfil Equation (6), then this first part of equation is equal to zero and **ε** = **x_r_**.

Observing the residual deviations signal Equation (9) can reveal unacceptable deviation characteristics and provide descriptive time information. Comparing the residual deviation signals of different observations can help to determine whether certain unacceptable deviations are typical for certain improperly performed swings.

## 3. Golf Swing Leading Arm Angular Velocity and Acceleration Measurements

Considering the different rotational and translational motion of involved body segments, the golf swing can be designated into multiple phases. The swing begins with the golf ball address, where the golfer's body is positioned parallel to the target line and the bottom of the clubhead is parallel to the ground. During the backswing, the golf club is raised away from the target to its final point, called the top of the backswing. This step is followed by the downswing, where the club is brought down to the impact and the golf club strikes the ball. The follow-through continues the motion of the club after the impact. During all of these phases, every involved body segment must move in a suitable manner to achieve a proper swing motion and successful shot.

### 3.1. Measurement Setup and Procedure

The swing data analysed in this research were obtained using a specifically designed wearable three-dimensional (3D) gyroscope and accelerometer motion sensor weighing 30 g and measuring 72 × 57 × 15 mm. The ITG3200-3 gyroscope is manufactured by InvenSense (Sunnyvale, CA, USA) and consists of three independent vibratory MEMS gyroscopes, while the MEMS three-axis liner accelerometer LIS331HH is manufactured by STMicroelectronics (Geneva, Switzerland). The full-scale range of the gyroscopes is ±2,000 °/s, while the dynamic accelerometer (which is user selectable) was set to ±24 g. Both devices operate on an ultra-low power supply (DC supply voltage between 2.1 V and 3.6 V for the gyroscope and 2.16 V and 3.6 V for the accelerometer), providing a 16-bit data output at a rate of 1,000 Hz that was stored on an internal memory card.

**Figure 1.**
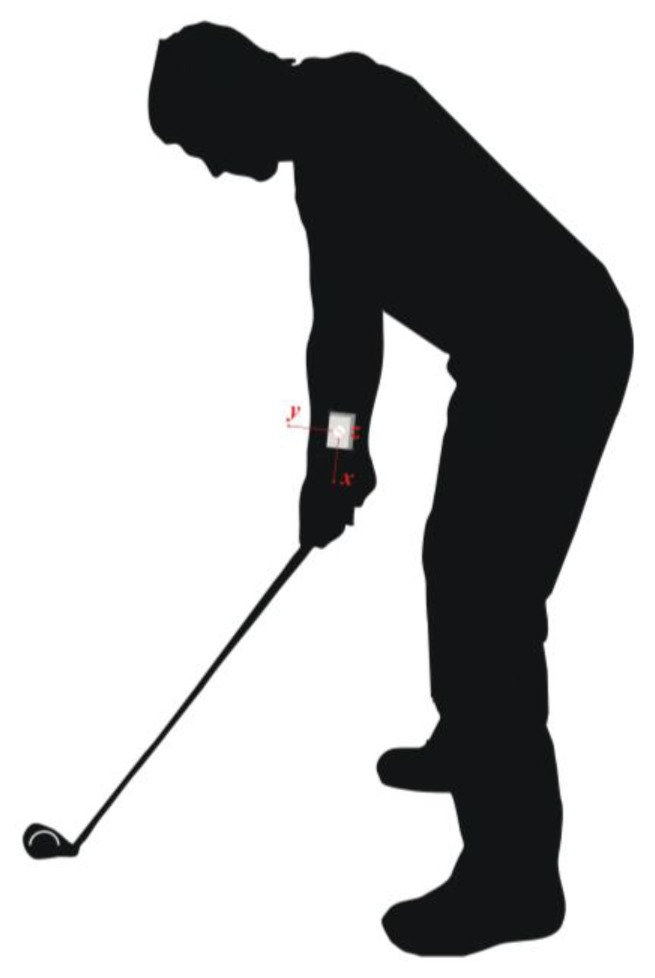
Position of the measurement sensor on the right-handed golfer's leading arm during the golf ball address.

**Figure 2.**
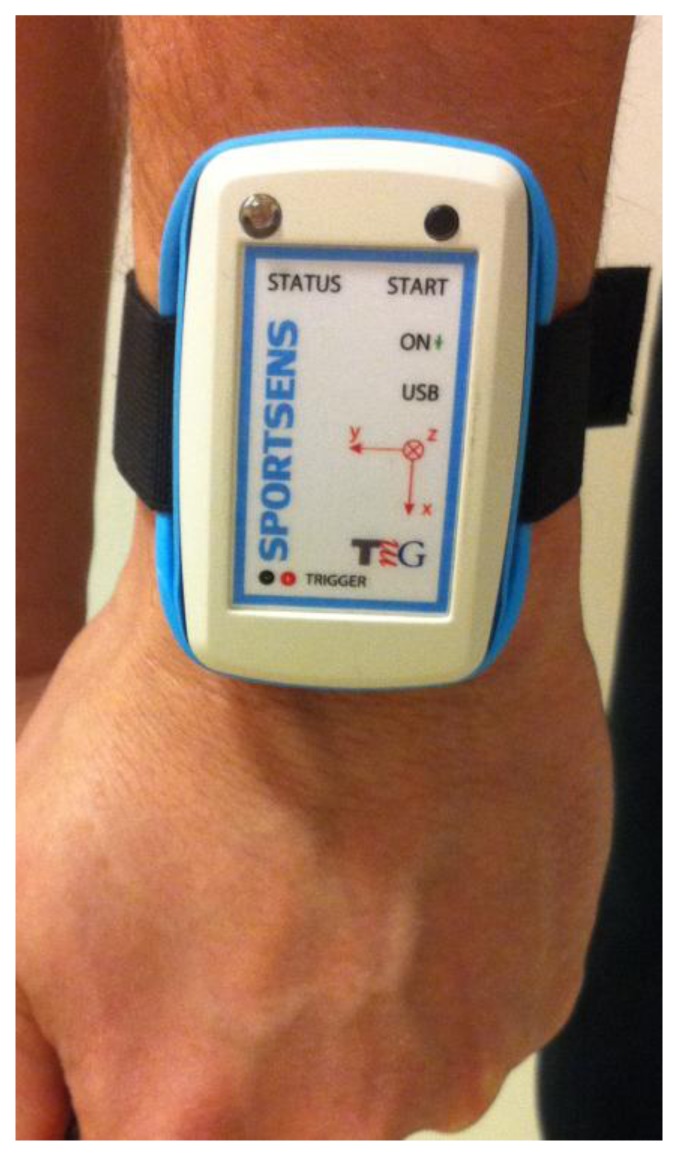
The specifically designed wearable 3D accelerometer and gyroscope motion sensor with its intrinsic coordinate system axis.

To capture the motion of the golfer's leading arm body segment (*i.e.*, left arm for a right-handed golfer), the sensor was positioned as illustrated in Figure 1. With such a position, the sensor-intrinsic x axis coincided with the longitudinal axis of the golfers leading arm, while the y and z axes referred to the lateral and vertical arm axes, respectively, as shown in Figure 2. As these axes are sensor intrinsic, they move together with the golfer's arm during the swing.

Measurements were performed on a 27-year-old right-handed professional player (weighing 74 kg and measuring 186 cm in height) using a hybrid golf club with an 18° loft in an outside environment during a clear day with neglectable wind. With the attached sensor in operation, the golfer was asked to shoot at a straight-line target at a specific distance as consistently as possible. The target was chosen to be at the average successful shot distance for a specific golfer using the particular club established prior to the measurements. The golfer performed in total 20 swings.

### 3.2. Swing Observation Labels

According to the measurement protocol, all performed swings were labelled with the direct outcome measures of the golf ball's flight direction and carry distance. The golfer also provided a subjective evaluation of each swing. These labels and evaluations were used to determine the overall golf swing performance. For a particular golf swing to be considered proper, the resulting shot had to be accurate and the subjective evaluation had to provide a satisfactory report. A shot was considered accurate if the ball's final distance from the target was no more than 1.5% of the expected carry distance. This concrete value was determined for a specific golfer by considering his skill level. If a golfer were less proficient, then a larger acceptable distance from the target would still qualify the shot as accurate. Analysed observations of improperly performed swings referred to any swing with at least one of the labels or evaluation mark considered unsatisfactory (*i.e.*, golf ball flight direction, carry distance, or subjective evaluation). 15 swings satisfied all requirements and were reported proper. Out of these 15, 10 were evaluated as ideal by the golfer himself.

### 3.3. Swing Data Extraction

The acquired swing data were pre-processed and analysed using custom-designed software in MATLAB (The Mathworks, Inc., Natick, MA, USA). The data were downsampled by 25/8 to obtain *f_s_* = 320 Hz digital signals. The output data were suitably corrected by subtracting the offset and linear time drift effects of the gyroscope and accelerometer.

Swing sequences were recognised utilising the following procedure. From the measured accelerations *a_x_*, *a_y_*, and *a_z_*, the acceleration magnitude was calculated. Based on these data, one 800-sample-long swing sequence was extracted by hand. The remaining swings were extracted and aligned considering the extreme values of the cross-correlation function of the manually obtained swing and the remaining acceleration magnitude data.

**Figure 3.**
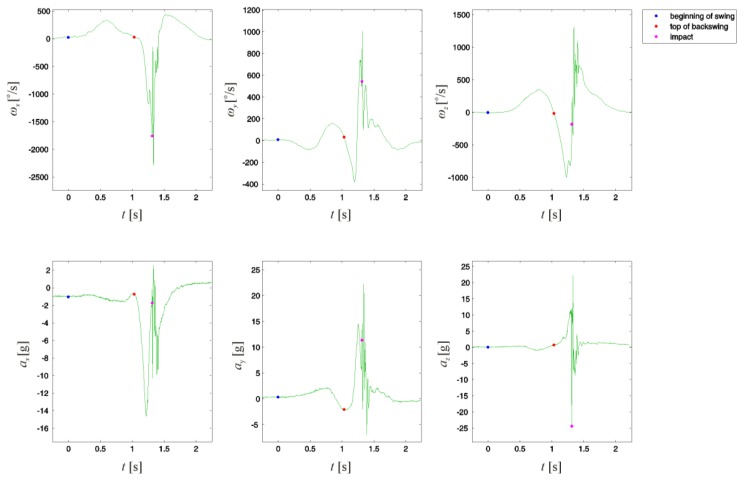
Golfer's leading arm angular velocities *ω_x_*, *ω_y_*, and *ω_z_* and accelerations *a_x_*, *a_y_*, and *a_z_* for one extracted swing sequence obtained using a wearable 3D motion sensor. The sensor-intrinsic x axis coincided with the longitudinal axis of the golfer's leading arm, while the y and z axes referred to the lateral and vertical arm axes, respectively.

The obtained leading arm angular velocities *ω_x_*, *ω_y_*, and *ω_z_* and accelerations *a_x_*, *a_y_*, and *a_z_* for one extracted swing sequence are illustrated in Figure 3. The beginning of the swing is determined as the point where the golfer's arm stillness turns to motion. The top of the backswing is considered as the point where the arm angular velocity magnitude was closest to reaching its zero value in a specified region of samples. Due to the reaction forces present at the impact of the clubhead with the ball, this point was determined as the point where acceleration magnitude reached its maximum value.

## 4. Results of the Improper Golf Swing Motion Detection

Given the reactive character of the golf swing, rather than exploring the entire swing sequence from golf ball address to the final point of the follow-through, focus was given to the golf backswing to obtain early detection of an improper swing motion. For this reason, we only analysed the first 0.625 s of the swing, *i.e.*, the first *L* = 200 backswing samples.

The analysis was separately performed on each of the six collected signals *ω*_x_, *ω*_y_, *ω*_z_, *a*_x_, *a*_y_, and *a*_z_. However, the best results were obtained for the angular velocity *ω*_x_. For simplicity, we present only these demonstrative results.

**Figure 4.**
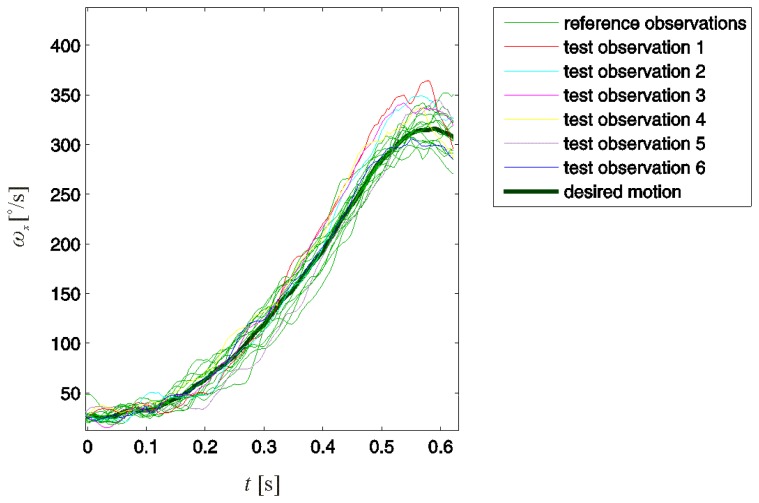
Reference and test observations of the golfer's leading arm rotation around its intrinsic longitudinal axis during the first 0.625 s of the backswing. All reference observations refer to properly performed swings. Test observations 1–5 refer to an improperly performed swing, and 6 refers to a properly performed swing. The desired motion is obtained as the mean of the reference observations.

Fourteen (*N* = 14) observations of swings evaluated as properly performed were included in the reference observation set. Observations of six additional swings, five improperly and one properly performed, were tested for improper motion. These test observations are denoted with numbers from one to six, where the first five refer to swings evaluated as improperly performed and six refers to a properly performed swing. The reference and test observations are presented in Figure 4.

Note that not all improper swing motions could be detected by directly comparing the test and reference observations. Test observations 1, 2, and 3 in Figure 4 could eventually be detected. However, test observations 4 and 5, although referring to improperly performed swings, could not be distinguished from the reference observations.

**Figure 5.**
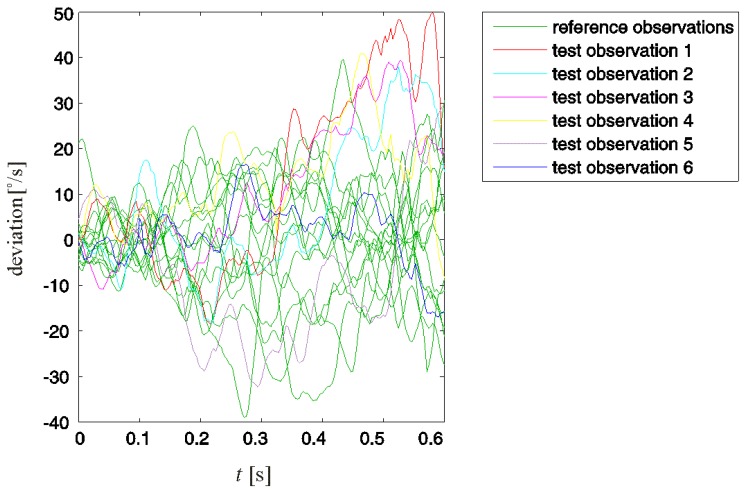
Acceptable and test deviations from the desired swing motion.

We performed the detection of an improper motion according to the procedure presented in Section 2. From the 14 reference observations, we obtained the desired motion **d** and the acceptable deviations set **R**. Test deviations **x***_i_* (1< *i* ≤6) represent deviations of test observations from the desired motion **d.** The acceptable deviations and test deviations are presented in Figure 5.

We tested deviations **x***_i_* for an improper motion following the three steps of the analysis.

### Step One

To test if Equation (4) is fulfilled, we obtained the maximal acceptable msv of reference deviations for the chosen tolerance factor *k* = 1.5 (The empirically chosen tolerance factor *k* = 1.5 yielded good results, even for cases not presented in this paper):

(10) $$k\cdot \max(\text{msv}({\text{r}}_{n}))=5.42\cdot 10^{2}.$$

The msv of **x***_i_* are presented in Table 1.

As only msv(**x**_1_) exceeded the maximal acceptable value, improper motion was only detected for test swing 1.

### Step Two

**Figure 6.**
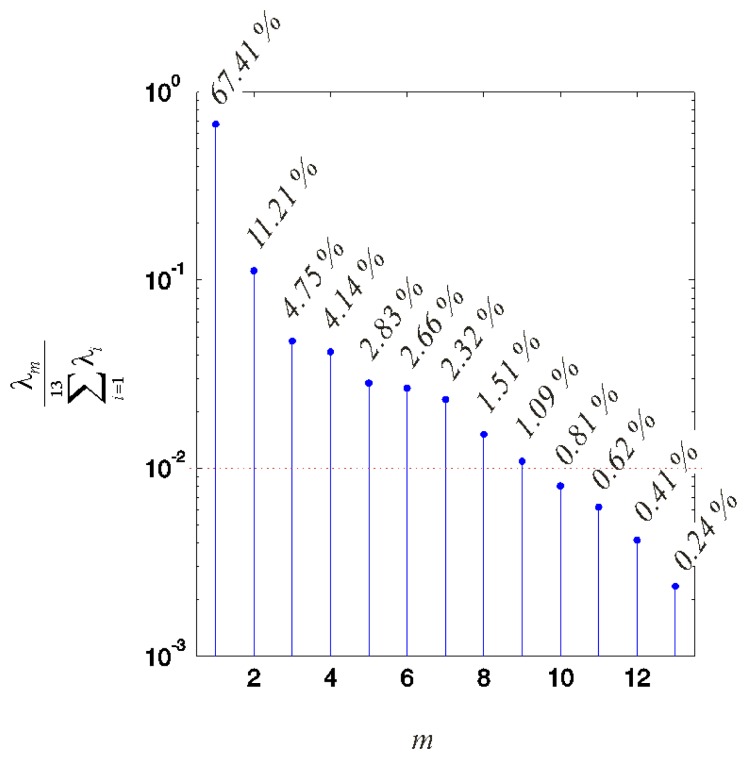
The variance of the acceptable deviations from the desired motion covered with the obtained principal components in logarithmic scale. The last four components each cover less than 1% of the variance. These components, covering 2.08% of the variance, were attributed to noise and/or different artefacts and teased from the first nine components. In sum, the first nine components cover the remaining 97.02% of the variance.

Following the procedure described in Section 2.2, we initially obtained 13 principal components **p***_m_* of **R**. The variance of **R** covered with each of these components is illustrated in Figure 6 in logarithmic scale.

The first *M* = 9 principal components were teased from the last four components attributed to noise and/or different artefacts.

**Figure 7.**
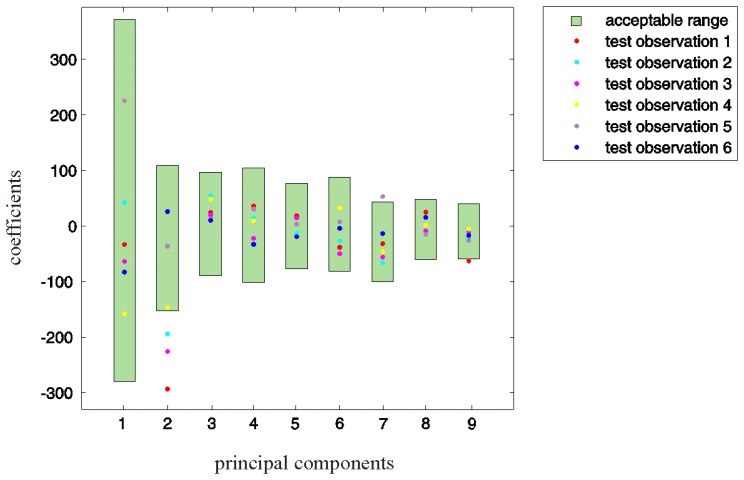
PCA coefficients of the test deviations from the desired swing motion. For test observations 1, 2, 3, and 5, at least one coefficient exceeds the acceptable range, indicating improper motion in the associated swings.

The PCA coefficients of the test deviations obtained according to Equation (5) are shown in Figure 7. By observing the concrete values, we can assert that for test observations 1, 2, 3, and 5, demand Equation (6) is not fulfilled for at least one reference principal component. This finding indicates an improper motion in the associated swings 1, 2, 3, and 5.

### Step Three

The first two steps of the analysis did not show an improper motion in two of the test observations, 4 and 6. We then examined the remaining deviation contents of all test observations.

We obtained the maximal acceptable msv of the residual deviations as:

(11) $$k\max(\text{msv}({\text{e}}_{n}))=11.53$$

The msv of **x_r_***_,i_* are presented in Table 2.

By observing the concrete values, we can assert that for test observations 1 to 5, the Equation (8) is not fulfilled. In addition to the already evaluated improper swings, this finding further indicates an improper motion in test observation 4.

These three steps show an improper motion for all five observations referring to improperly performed swings. By subtracting the established desired swing motion and acceptable deviations from all observations, we acquired the associated residual deviations. For reference observations, these deviations are the column vectors **e***_n_* of **E** obtained according to Equation (3). For test observations, these vectors are denoted with **ε***_i_* and obtained according to Equation (9).

**Figure 8.**
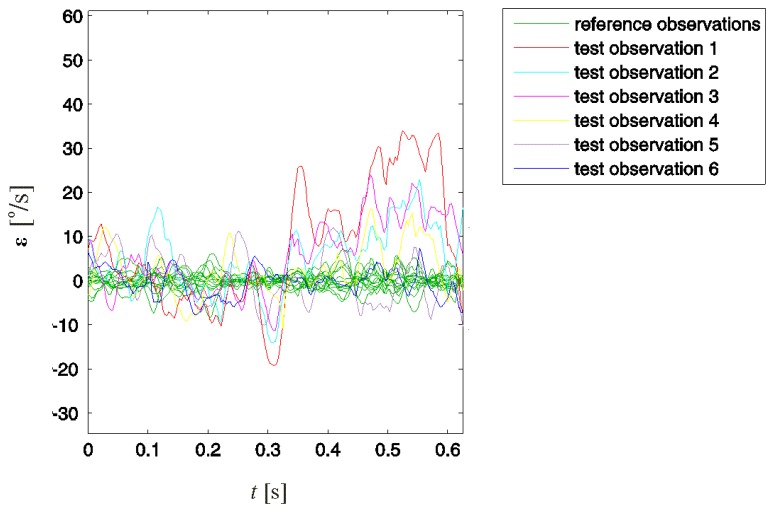
Acceptable and test observation residual deviations in time domain. Acceptable deviations residuals represent deviations in properly performed swings attributed to noise and/or different artefacts. The deviation residuals for test observations 1–5, for which improper motion was detected, considerably exceed acceptable deviations residuals. Consistently positive values in the second half of the considered swing interval for test observations 1–4 indicate a typical improper motion in the associated swings.

Observing the residual deviations (Figure 8) provided a better distinction of improper motion than observing the original observations (Figure 4). For all five test observations in which improper motion was detected, the residual deviations at some point considerably exceeded the values of reference observations. In test observations 1–4, the consistent presence of positive offset deviations in the second half of the considered backswing interval is observable. This similar behaviour of **ε**_1_–**ε**_4_ indicates a typical improper motion in the associated swings. Considering that we analysed angular velocity *ω*_x_, this typical improper motion can be attributed to excessive rotation of the leading arm around its intrinsic longitudinal axis in this specific part of the backswing.

## 5. Conclusions

This paper presented an analysis of a golf swing for the early detection of an improper swing motion using wearable 3D motion sensors. The analysis was performed by exploring motion deviations from the desired swing motion.

Measurements were performed on a professional golfer using a hybrid club. The wearable 3D motion sensor, comprised of gyroscopes and accelerometers, was positioned on the golfer's leading arm. All swing observations were labelled according to the golf ball's flight direction and carry distance, as well as subjective evaluations provided by the golfer. These labels were used to evaluate the performance of the golf swing.

Fourteen observations of swings evaluated as properly performed were included in the reference set. These observations were used to determine the desired swing motion and principal components of the acceptable swing motion deviations. Using these components, motion deviations in six additional swing observations were examined. The results obtained for the angular velocity, representing the golfer's leading arm rotation around its intrinsic longitudinal axis, showed that an improper motion can be detected in the early phase of the swing, *i.e.*, in the first 0.625 s of the backswing.

Additional analysis of the residual deviations in the time domain provided a better understanding of the improper swing motion.

The analysis was performed under the assumption that the reference set of properly performed swings encompassed all acceptable deviations of a proper golf swing motion. Because of this assumption, properly performed swings that were falsely evaluated as improperly performed had to be included in the reference observation set.

The method was applied to a particular swing with a particular club and in a particular environment. For a different club, shot or environment, a different reference set of properly performed swings would have to be constructed.

Using the proposed analysis, swing performance evaluation was achieved on an individual basis, and the presence of an improper motion was explored with reference to that player's properly performed swings.

The presented analysis can be used as a valuable tool in the performance improvement process adapted to a golfer's individual specifics. During the improvement process, the reference set can be rebuilt with tighter margins of labelling a swing as proper.

For each analysed unsuccessful shot, the player can be given information about which performance deviations, characterised as improper motions, preceded and likely caused the improper golf ball impact.

Due to the importance of the golf swing tempo, all reference swings should have the same timing. If the timing of the analyzed swing would differ from the timing of the reference swings, the method applied would detect improper motion. This would then enable the player to adjust to a proper, repeatable golf swing tempo.

In the future, we will try to test the method proposed on a significantly larger database, which will include different shots with different clubs for different players. However, building such a database could last up to a year or even more. Further study can address multiple sensor usage, enabling motion data capture of different body parts and exploring their mutual relation and possibilities for sensor fusion.

Exploring the possibilities of developing biofeedback applications relying on early-phase improper motion detection for real-time golf motion supervision and training can also motivate further study. Providing efficient swing analysis and performance evaluation in real time and offering immediate information on the likely outcome of the performing golf swing could potentially transform the approach to golf instruction and practice.

## Figures and Tables

**Table 1.** Mean square values (msv) of the test deviations x*_i_* from the desired swing motion. Only msv(x_1_) exceeds the maximal acceptable value of 5.42 × 10^2^, indicating improper motion in test swing 1.

*i*	**msv(x***_i_***)**
1	5.53 × 10^2^
2	2.83 × 10^2^
3	3.31 × 10^2^
4	2.89 × 10^2^
5	3.15 × 10^2^
6	0.60 × 10^2^

**Table 2.** The mean square values (msv) of the test deviation remaining content x_r,_*_i_* not covered with the reference principal components. For test observations 1-5, msv(x_r,_*_i_*) exceeds the acceptable value of msv = 11.53, indicating an improper motion in the associated swings 1–5.

***i***	**msv(x_r,_***_i_***))**
1	73.40
2	40.19
3	22.75
4	27.35
5	31.02
6	11.15
